# Supplementary material for: Influence of reduced graphene oxide addition on kerf width in abrasive water jet machining of nanofiller added epoxy-glass fibre composite
Source: PLoS One. 2022 Aug 16;17(8):e0270505. doi: 10.1371/journal.pone.0270505 (PMC9380911; doi:10.1371/journal.pone.0270505)
Supplement: S1 File — (DOCX) [file pone.0270505.s001.docx]

**Influence of reduced graphene oxide addition on kerf width in abrasive water jet machining of nanofiller added Epoxy-Glass Fibre composite**

**Kavimani. V^1^, Gopal. P. M^1^, Stalin. B^2^, Balasubramani. V^3^, Dhinakaran. V^4^, Nagaprasad. N^5^, Leta Tesfaye Jule^6,7^, Krishnaraj Ramaswamy^6,8,*^**

^1^Centre for Material Science, Department of Mechanical Engineering, Karpagam Academy of Higher Education, Coimbatore, Tamil Nadu, India.

^2^Department of Mechanical Engineering, Anna University, Regional Campus Madurai, Madurai, Tamil Nadu 625019, India.

^3^Department of Mechanical Engineering, Thiagarajar College of Engineering, Madurai-625015, Tamil Nadu, India.

^4^Department of Mechanical Engineering, Chennai Institute of Technology, Kundrathur, Chennai 600 069, Tamil Nadu, India.

^5^Department of Mechanical Engineering, ULTRA College of Engineering and Technology, Madurai – 625 104, Tamil Nadu, India.

^6^Centre for Excellence-Indigenous Knowledge, Innovative Technology Transfer and Entrepreneurship, DambiDollo University, Ethiopia.

^7^Department of Physics, DambiDollo University, Ethiopia.

^8^Department of Mechanical Engineering, DambiDollo University, Ethiopia.

***Corresponding Author. Tel: +251946381416.

*Email address:* prof.dr.krishnaraj@dadu.edu.et (RK)

**Supplementary data**

**S1 Table.** Matrix scatterplot of L27 OA dataset& Hierarchical clustering of correlation coefficient matrix of input variables

| PP | TS | SOD | 0 KW | 0.1 KW | 0.2KW | 0.3KW | SN0 | SN0.1 | SN0.2 | SN0.3 |
| --- | --- | --- | --- | --- | --- | --- | --- | --- | --- | --- |
| 220 | 30 | 1 | 1.46 | 1.41 | 1.5 | 1.61 | -3.28706 | -2.98438 | -3.52183 | -4.13652 |
| 220 | 30 | 2 | 1.51 | 1.49 | 1.56 | 1.67 | -3.57954 | -3.46373 | -3.86249 | -4.45433 |
| 220 | 30 | 3 | 1.56 | 1.54 | 1.64 | 1.75 | -3.86249 | -3.75041 | -4.29688 | -4.86076 |
| 220 | 40 | 1 | 1.43 | 1.39 | 1.48 | 1.57 | -3.10672 | -2.8603 | -3.40523 | -3.91799 |
| 220 | 40 | 2 | 1.49 | 1.48 | 1.54 | 1.61 | -3.46373 | -3.40523 | -3.75041 | -4.13652 |
| 220 | 40 | 3 | 1.56 | 1.52 | 1.59 | 1.72 | -3.86249 | -3.63687 | -4.02794 | -4.71057 |
| 220 | 50 | 1 | 1.37 | 1.35 | 1.4 | 1.52 | -2.73441 | -2.60668 | -2.92256 | -3.63687 |
| 220 | 50 | 2 | 1.41 | 1.4 | 1.46 | 1.59 | -2.98438 | -2.92256 | -3.28706 | -4.02794 |
| 220 | 50 | 3 | 1.52 | 1.48 | 1.57 | 1.67 | -3.63687 | -3.40523 | -3.91799 | -4.45433 |
| 240 | 30 | 1 | 1.52 | 1.51 | 1.55 | 1.67 | -3.63687 | -3.57954 | -3.80663 | -4.45433 |
| 240 | 30 | 2 | 1.55 | 1.52 | 1.59 | 1.67 | -3.80663 | -3.63687 | -4.02794 | -4.45433 |
| 240 | 30 | 3 | 1.64 | 1.61 | 1.67 | 1.8 | -4.29688 | -4.13652 | -4.45433 | -5.10545 |
| 240 | 40 | 1 | 1.49 | 1.46 | 1.53 | 1.58 | -3.46373 | -3.28706 | -3.69383 | -3.97314 |
| 240 | 40 | 2 | 1.51 | 1.48 | 1.55 | 1.66 | -3.57954 | -3.40523 | -3.80663 | -4.40216 |
| 240 | 40 | 3 | 1.63 | 1.6 | 1.69 | 1.79 | -4.24375 | -4.0824 | -4.55773 | -5.05706 |
| 240 | 50 | 1 | 1.43 | 1.39 | 1.47 | 1.57 | -3.10672 | -2.8603 | -3.34635 | -3.91799 |
| 240 | 50 | 2 | 1.49 | 1.47 | 1.53 | 1.64 | -3.46373 | -3.34635 | -3.69383 | -4.29688 |
| 240 | 50 | 3 | 1.59 | 1.56 | 1.64 | 1.75 | -4.02794 | -3.86249 | -4.29688 | -4.86076 |
| 260 | 30 | 1 | 1.49 | 1.46 | 1.52 | 1.63 | -3.46373 | -3.28706 | -3.63687 | -4.24375 |
| 260 | 30 | 2 | 1.62 | 1.57 | 1.65 | 1.75 | -4.1903 | -3.91799 | -4.34968 | -4.86076 |
| 260 | 30 | 3 | 1.66 | 1.64 | 1.72 | 1.85 | -4.40216 | -4.29688 | -4.71057 | -5.34343 |
| 260 | 40 | 1 | 1.49 | 1.45 | 1.52 | 1.64 | -3.46373 | -3.22736 | -3.63687 | -4.29688 |
| 260 | 40 | 2 | 1.56 | 1.52 | 1.58 | 1.67 | -3.86249 | -3.63687 | -3.97314 | -4.45433 |
| 260 | 40 | 3 | 1.66 | 1.6 | 1.73 | 1.84 | -4.40216 | -4.0824 | -4.76092 | -5.29636 |
| 260 | 50 | 1 | 1.44 | 1.4 | 1.47 | 1.56 | -3.16725 | -2.92256 | -3.34635 | -3.86249 |
| 260 | 50 | 2 | 1.53 | 1.49 | 1.56 | 1.68 | -3.69383 | -3.46373 | -3.86249 | -4.50619 |
| 260 | 50 | 3 | 1.61 | 1.58 | 1.63 | 1.74 | -4.13652 | -3.97314 | -4.24375 | -4.81098 |

**S2 Table.** Effect of machining parameter on Kerf width of developed composite

| 0 wt.% r-Go | 0.1 wt.% r-GO | 0.2wt .%r- GO | 0.3 wt.% r-GO |
| --- | --- | --- | --- |
| 1.46 | 1.41 | 1.50 | 1.61 |
| 1.51 | 1.49 | 1.56 | 1.67 |
| 1.56 | 1.54 | 1.64 | 1.75 |
| 1.43 | 1.39 | 1.48 | 1.57 |
| 1.49 | 1.48 | 1.54 | 1.61 |
| 1.56 | 1.52 | 1.59 | 1.72 |
| 1.37 | 1.35 | 1.40 | 1.52 |
| 1.41 | 1.40 | 1.46 | 1.59 |
| 1.52 | 1.48 | 1.57 | 1.67 |
| 1.52 | 1.51 | 1.55 | 1.67 |
| 1.55 | 1.52 | 1.59 | 1.67 |
| 1.64 | 1.61 | 1.67 | 1.80 |
| 1.49 | 1.46 | 1.53 | 1.58 |
| 1.51 | 1.48 | 1.55 | 1.66 |
| 1.63 | 1.60 | 1.69 | 1.79 |
| 1.43 | 1.39 | 1.47 | 1.57 |
| 1.49 | 1.47 | 1.53 | 1.64 |
| 1.59 | 1.56 | 1.64 | 1.75 |
| 1.49 | 1.46 | 1.52 | 1.63 |
| 1.62 | 1.57 | 1.65 | 1.75 |
| 1.66 | 1.64 | 1.72 | 1.85 |
| 1.49 | 1.45 | 1.52 | 1.64 |
| 1.56 | 1.52 | 1.58 | 1.67 |
| 1.66 | 1.60 | 1.73 | 1.84 |
| 1.44 | 1.40 | 1.47 | 1.56 |
| 1.53 | 1.49 | 1.56 | 1.68 |
| 1.61 | 1.58 | 1.63 | 1.74 |

**S3 Table.** Contribution of machining parameters in 0.1 wt. % filler loaded composite

| Condition | 0 wt.% r-Go | 0.1 wt.% r-GO | 0.2wt .%r- GO | 0.3 wt.% r-GO |
| --- | --- | --- | --- | --- |
| Pump Pressure | 20.94899 | 17.73324 | 13.91118 | 12.1524 |
| Transverse speed | 14.11965 | 14.74451 | 14.95175 | 12.94675 |
| Stand of distance | 60.55164 | 63.00719 | 65.82698 | 69.40475 |
