# Supplementary material for: Influence of reduced graphene oxide addition on kerf width in abrasive water jet machining of nanofiller added epoxy-glass fibre composite
Source: PLoS One. 2022 Aug 16;17(8):e0270505. doi: 10.1371/journal.pone.0270505 (PMC9380911; doi:10.1371/journal.pone.0270505)
Supplement: Attachment — Submitted filename: Comments.docx [file pone.0270505.s002.docx]

Comments and Suggestions for Authors

The manuscript “Influence of reduced graphene oxide addition on kerf width in abrasive water jet machining of nano filler added Epoxy-Glass Fibre composite” by Krishnaraj et.al. present novel and interesting results that deserve to be published after several minor improvements:

1. It will be of great interest if the Surface roughness at varied Water Jet Pressure and material removal rate at different traverse speed should be included in detail with proper interpretation of results.
2. Scanning electron microscope is the most important testing technique to understand the distribution of nanoparticles as well the surface information. The results are missing, and it should be included in it.
3. Mechanical testing in combinations for detecting flexural, impact, tensile, density and hardness properties should be included as well.
4. Rheological properties are important in order to understand the nanofiller and epoxy blends. Please included these properties.
5. The Figure captions are much unsatisfactory. It is a fundamental responsibility of the authors to make Figures self-explanatory. Go to the guidelines and examples and prepare the Figure captions accordingly. This applies to all Figures with this manuscript.
6. FTIR data should be included.
