# Supplementary material for: Influence of reduced graphene oxide addition on kerf width in abrasive water jet machining of nanofiller added epoxy-glass fibre composite
Source: PLoS One. 2022 Aug 16;17(8):e0270505. doi: 10.1371/journal.pone.0270505 (PMC9380911; doi:10.1371/journal.pone.0270505)
Supplement: Attachment — Submitted filename: Response to reviewer.docx [file pone.0270505.s003.docx]

**Response to reviewer**:

**Reviewer 1**

***Comment 1: It will be of great interest if the Surface roughness at varied Water Jet Pressure and material removal rate at different traverse speed should be included in detail with proper interpretation of results.***

***Response: Thanks for your valuable comments. This article is mainly focus on effect of machining parameters on kerf width here the other output parameter are neglected. The parameter such as surface roughness and material removal rate are optimized and interpreted as separate manuscript.***

***Comment 2: Scanning electron microscope is the most important testing technique to understand the distribution of nanoparticles as well the surface information. The results are missing, and it should be included in it.***

***Response: presence of filler has been confirmed by XRD analysis it is already published in our article .<https://www.hindawi.com/journals/apt/2021/6627743/>***

***Comment 3: Mechanical testing in combinations for detecting flexural, impact, tensile, density and hardness properties should be included as well.***

***Response: Thanks for your valuable suggestion. The mechanical properties of the developed r-GO-MMT reinforced composite have been already published in our article. Please refer the link .<https://www.hindawi.com/journals/apt/2021/6627743/>.The necessary citation has been made in this revised manuscript***

These developed composite exhibits better mechanical and flame retardancy properties upto 0.3 wt. % addition of r-GO fillers. The detailed investigation on mechanical behaviour of developed composite was illustrated in our previous report [29].

***Comment 4: Rheological properties are important in order to understand the nanofiller and epoxy blends. Please include these properties.***

***Response: This manuscript is extensively focus on machinability of developed composite hence rheological studies may be neglected***

***Comment 5: The Figure captions are much unsatisfactory. It is a fundamental responsibility of the authors to make Figures self-explanatory. Go to the guidelines and examples and prepare the Figure captions accordingly. This applies to all Figures with this manuscript.***

***Response: As per reviewer recommendation captions of the figure has been modified***

***Comment 6 : FTIR data should be included.***

***Response: Fundamental characterization has been made in our previous manuscript and the necessary citation has been made.***

**Reviewer 2**

***Comments 1) provide the L27 experiments combinations.***

***Response: As per reviewer suggestion L27 OA has been added in this revised manuscript.***

| A | B | C | **Kerf width (µm)** | | | | **S/N ratio** | | | |
| --- | --- | --- | --- | --- | --- | --- | --- | --- | --- | --- |
|  |  |  | **0%** | **0.1 %** | **0.2%** | **0.3%** | **0%** | **0.1 %** | **0.2%** | **0.3%** |
| 220 | 30 | 1 | 1.46 | 1.41 | 1.50 | 1.61 | -3.29 | -2.98 | -3.52 | -4.14 |
| 220 | 30 | 2 | 1.51 | 1.49 | 1.56 | 1.67 | -3.58 | -3.46 | -3.86 | -4.45 |
| 220 | 30 | 3 | 1.56 | 1.54 | 1.64 | 1.75 | -3.86 | -3.75 | -4.30 | -4.86 |
| 220 | 40 | 1 | 1.43 | 1.39 | 1.48 | 1.57 | -3.11 | -2.86 | -3.41 | -3.92 |
| 220 | 40 | 2 | 1.49 | 1.48 | 1.54 | 1.61 | -3.46 | -3.41 | -3.75 | -4.14 |
| 220 | 40 | 3 | 1.56 | 1.52 | 1.59 | 1.72 | -3.86 | -3.64 | -4.03 | -4.71 |
| 220 | 50 | 1 | 1.37 | 1.35 | 1.40 | 1.52 | -2.73 | -2.61 | -2.92 | -3.64 |
| 220 | 50 | 2 | 1.41 | 1.40 | 1.46 | 1.59 | -2.98 | -2.92 | -3.29 | -4.03 |
| 220 | 50 | 3 | 1.52 | 1.48 | 1.57 | 1.67 | -3.64 | -3.41 | -3.92 | -4.45 |
| 240 | 30 | 1 | 1.52 | 1.51 | 1.55 | 1.67 | -3.64 | -3.58 | -3.81 | -4.45 |
| 240 | 30 | 2 | 1.55 | 1.52 | 1.59 | 1.67 | -3.81 | -3.64 | -4.03 | -4.45 |
| 240 | 30 | 3 | 1.64 | 1.61 | 1.67 | 1.80 | -4.30 | -4.14 | -4.45 | -5.11 |
| 240 | 40 | 1 | 1.49 | 1.46 | 1.53 | 1.58 | -3.46 | -3.29 | -3.69 | -3.97 |
| 240 | 40 | 2 | 1.51 | 1.48 | 1.55 | 1.66 | -3.58 | -3.41 | -3.81 | -4.40 |
| 240 | 40 | 3 | 1.63 | 1.60 | 1.69 | 1.79 | -4.24 | -4.08 | -4.56 | -5.06 |
| 240 | 50 | 1 | 1.43 | 1.39 | 1.47 | 1.57 | -3.11 | -2.86 | -3.35 | -3.92 |
| 240 | 50 | 2 | 1.49 | 1.47 | 1.53 | 1.64 | -3.46 | -3.35 | -3.69 | -4.30 |
| 240 | 50 | 3 | 1.59 | 1.56 | 1.64 | 1.75 | -4.03 | -3.86 | -4.30 | -4.86 |
| 260 | 30 | 1 | 1.49 | 1.46 | 1.52 | 1.63 | -3.46 | -3.29 | -3.64 | -4.24 |
| 260 | 30 | 2 | 1.62 | 1.57 | 1.65 | 1.75 | -4.19 | -3.92 | -4.35 | -4.86 |
| 260 | 30 | 3 | 1.66 | 1.64 | 1.72 | 1.85 | -4.40 | -4.30 | -4.71 | -5.34 |
| 260 | 40 | 1 | 1.49 | 1.45 | 1.52 | 1.64 | -3.46 | -3.23 | -3.64 | -4.30 |
| 260 | 40 | 2 | 1.56 | 1.52 | 1.58 | 1.67 | -3.86 | -3.64 | -3.97 | -4.45 |
| 260 | 40 | 3 | 1.66 | 1.60 | 1.73 | 1.84 | -4.40 | -4.08 | -4.76 | -5.30 |
| 260 | 50 | 1 | 1.44 | 1.40 | 1.47 | 1.56 | -3.17 | -2.92 | -3.35 | -3.86 |
| 260 | 50 | 2 | 1.53 | 1.49 | 1.56 | 1.68 | -3.69 | -3.46 | -3.86 | -4.51 |
| 260 | 50 | 3 | 1.61 | 1.58 | 1.63 | 1.74 | -4.14 | -3.97 | -4.24 | -4.81 |

***Comments 2) Provide the measured readings of the kerf in a table with the instrument used for the measurement of kerf.***

***Response: As per reviewer console, the kerf width measurement has been detailed in this revised manuscript.***

Kerf width is taken as the output parameter and it is measured by calculating the distance between machined slots using Leica microscope coupled with image analyser as depicted in figure 2b.

***Comments 3) Figure 2 and Figure 3 should be explained in detail with their relevance.***

***Response: Necessary correction has been made as per reviewer recommendation***

Scatter-plot matrix is used to identify the correlation among the set of variable pairs. These pair wise correlations can be organizes in a form of matrix. In general present matrix pair in diagonal order depicts the better correlation among the matrix pairs and absents of outliers in the attained output data’s. Hierarchical cluster is an analysis worked by algorithm in which the similar data’s are grouped in a form of clusters. Herein each clusters are varied from each other and the values are widely similar to each other. From figure 4 it can be noted that the values of output parameter are grouped into four clusters with two expressive colour blue and brown (dark and lighter).These are order at the range of -1 to 1 with respect to input parameter. The values of output parameters are predominant ordering from top to bottom displayed in the form of lighter and dark colours of blue and brown.

***4) Fabricated composites pictures to be provided.***

***Response: as per reviewer recommendation pictures of fabricated composite has been added***

***Comments 5) Effect on the composites layers can be discussed during the cutting.***

***Response: This work is mainly focus on influence of machining parameter on kerf width hence effect of composite layers are neglected in this manuscript***
